# Expression of *Piwi*, *MMP*, *TIMP*, and *Sox* during Gut Regeneration in Holothurian *Eupentacta fraudatrix* (Holothuroidea, Dendrochirotida)

**DOI:** 10.3390/genes12081292

**Published:** 2021-08-23

**Authors:** Igor Yu. Dolmatov, Nadezhda V. Kalacheva, Ekaterina S. Tkacheva, Alena P. Shulga, Eugenia G. Zavalnaya, Ekaterina V. Shamshurina, Alexander S. Girich, Alexey V. Boyko, Marina G. Eliseikina

**Affiliations:** A.V. Zhirmunsky National Scientific Center of Marine Biology, Far Eastern Branch, Russian Academy of Sciences, Palchevsky 17, 690041 Vladivostok, Russia; loriel@list.ru (N.V.K.); estkacheva@gmail.com (E.S.T.); alena.lavruk@mail.ru (A.P.S.); Eugenia_94@inbox.ru (E.G.Z.); eshamshurina@rambler.ru (E.V.S.); astromoon@mail.ru (A.S.G.); alteroldis@gmail.com (A.V.B.); meliseikina@yandex.ru (M.G.E.)

**Keywords:** holothurian, regeneration, coelomocytes, Piwi, MMP, TIMP, tensilin, Sox

## Abstract

Mesodermal cells of holothurian *Eupentacta fraudatrix* can transdifferentiate into enterocytes during the regeneration of the digestive system. In this study, we investigated the expression of several genes involved in gut regeneration in *E. fraudatrix.* Moreover, the localization of progenitor cells of coelomocytes, juvenile cells, and their participation in the formation of the luminal epithelium of the digestive tube were studied. It was shown that Piwi-positive cells were not involved in the formation of the luminal epithelium of the digestive tube. *Ef-72 kDa type IV collagenase* and *Ef-MMP16* had an individual expression profile and possibly different functions. The *Ef-tensilin3* gene exhibited the highest expression and indicates its potential role in regeneration. *Ef-Sox9/10* and *Ef-Sox17* in *E. fraudatrix* may participate in the mechanism of transdifferentiation of coelomic epithelial cells. Their transcripts mark the cells that plunge into the connective tissue of the gut anlage and give rise to enterocytes. *Ef-Sox9/10* probably controls the switching of mesodermal cells to the enterocyte phenotype, while *Ef-Sox17* may be involved in the regulation of the initial stages of transdifferentiation.

## 1. Introduction

Echinoderms are known for their good regenerative abilities. They can regenerate both appendages (arms, tentacles, and tube feet) and internal organs [1,2]. During regeneration, the formation of lost structures is carried out due to dedifferentiated cells of the preserved tissues [2,3,4,5,6,7,8,9]. The presence of stem cells, with the exception of progenitor cells of coelomocytes [10,11] and primordial germ cells, has not been established by morphological methods [12,13]. In this regard, attempts are being made to detect echinoderm stem/progenitor cells using molecular markers. In holothurians, the expression of orthologs of genes *Lgr5* and *Bmi1* and Yamanaka factors (*Oct4*, *KLF4*, *Sox2*, and *myc*) was studied [14,15]. However, all of these genes, with the exception of *myc*, showed no significant activity in the regeneration of internal organs. Research has shown that, in holothurians, Myc is assumed to facilitate cell dedifferentiation and trigger programmed cell death [16].

One of the pluripotency markers is the Piwi protein [17,18,19]. Expression of the *Piwi* gene is characteristic not only of germline cells but also of multipotent and pluripotent stem cells in bilaterian animals with a high regenerative capacity [18,20,21]. *Piwi*-expressing somatic stem cells are involved in regeneration processes in some species, and the inhibition of their expression leads to defects in regeneration or its complete cessation and, as a consequence, to the death of the organism [22,23].

The *Piwi* and *Vasa* genes are expressed in various cells during regeneration in echinoderms [11,24,25,26]. However, whether these cells are stem cells, in most cases, has not been established. The only exception is the holothurian *E. fraudatrix* (D’yakonov & Baranova in D’yakonov, Baranova & Savel’eva 1958). In this species, the Piwi protein marks part of the population of progenitor cells of coelomocytes, juvenile cells [11]. However, the role of juvenile cells in the regeneration of internal organs has not been studied.

Among echinoderms, the mechanisms of regeneration have been thoroughly studied in holothurians [2,27,28,29,30]. In these animals, the morphological features of the formation of the digestive system, as well as the participation of a number of genes in the morphogenesis of the gut, have been studied in detail [28,30,31,32,33]. It was shown that extracellular matrix (ECM) remodeling plays an important role in the regeneration in holothurians. Blocking the activity of the matrix metalloproteinases (MMPs) led to slower gut growth [34,35]. In the holothurian *Apostichopus japonicus* (Selenka 1867), two *MMP* genes have been identified, the expression of which increase during regeneration [36]. In addition, a large number of genes for MMP inhibitors, *tissue inhibitor of matrix metalloprotease* (*TIMPs*), have been found in holothurians [37,38]. It has been shown that *TIMPs* are activated during asexual reproduction and regeneration [31,38]. However, their role in these processes has not been studied. There is a suggestion that one of the TIMP-like proteins, tensilin, may be involved in the mechanisms of changing the mechanical properties of the connective tissue of echinoderms [39].

One of the key regulators of endodermal cell differentiation and the formation of digestive organs in animals is the Sox gene family, particularly *Sox9* and *Sox17* [40,41]. In mammals, *Sox9* is a marker for adult and/or facultative stem cells in the intestines [42,43]. Genes *Sox9* and *Sox17* are also activated during the regeneration of various organs in vertebrates [42,44,45,46]. At the same time, their participation in gut regeneration in holothurians has not been studied.

Holothurian *E. fraudatrix* is interesting in that, during the regeneration of the anterior part of the digestive system, enterocytes are formed due to the transdifferentiation of coelomic epithelial cells [47]. This species is capable of evisceration (autotomy of internal organs) through the anterior end of the body, as a result of which, the animal loses its entire digestive system and the oral complex of the organs (aquapharyngeal bulb, AB) [48,49]. In *E. fraudatrix*, gut regeneration occurs, like in other holothurians, as a result of the formation and growth of two anlagen along the ventral edge of the intestinal mesentery [48,49]. The posterior one grows from the cloaca, and the anterior one grows from the regenerating AB. First, along the edge of the mesentery, anterior and posterior connective tissue thickenings develop. They represent the basis of the future digestive tube. Then, the luminal epithelium of the gut is formed in them. In the posterior anlage, the luminal epithelium arises due to the ingrowth of the cloacal luminal epithelium. In the anterior anlage, cells of endodermal origin are absent. Here, 5–7 days post-evisceration (dpe), the cells of the coelomic epithelium on the ventral side of the thickening begin to plunge into the connective tissue, undergo transdifferentiation, and are converted into enterocytes [47]. The general scheme of the formation of the anterior gut anlage in *E. fraudatrix* was presented in a recently published review [28].

To elucidate the molecular mechanisms of transdifferentiation, we analyzed the transcriptome of the anterior gut anlage in *E. fraudatrix* [31]. It has been shown that, during the regeneration of the digestive system, a large number of different genes are expressed. However, apart from some transcription factors, other differentially expressed genes (DEGs) have not been studied [31]. In this regard, we analyzed the *E. fraudatrix* transcriptome and studied the distribution of the transcripts of the genes of the MMP, TIMP, and Sox families, the expressions of which increase during transdifferentiation and the formation of the gut luminal epithelium. In addition, to study the question of the participation of stem cells in the regeneration of the digestive system in this species, the *Piwi* gene expression and the distribution of Piwi-positive cells in the tissues of *E. fraudatrix* were investigated.

## 2. Materials and Methods

### 2.1. Animal Collection, Maintenance, and Evisceration

Adult individuals of the holothurian *E. fraudatrix* (Holothuroidea, Dendrochirotida) were collected in the Peter the Great Bay, Sea of Japan and kept in 370 L tanks during one week with running aerated seawater at 16 °C without any feeding. Evisceration was induced by an injection of 2% KCl. Regenerating individuals were kept in the same tanks with aerated seawater. The water was changed daily. Most of the animals were taken for analysis during the formation of the anterior anlage and luminal epithelium of the gut after 5–7 and 10 dpe [31,47].

### 2.2. Real-Time PCR

Total RNA was isolated from *E. fraudatrix* digestive system anlages on 3, 5, 7, 10, 14, and 20 dpe using ExtractRNA (Evrogen). Five individuals were taken for each regeneration stage. Homogenization was carried out with metal balls on a TissueLyser LT homogenizer (Quagen, Germany). Total RNA was treated with DNase I (Thermo Scientific, Waltham, MA, USA) and purified by a GeneJet RNA Purification Kit (Thermo Scientific). Isolated RNA was analyzed using a BioSpec-nano spectrophotometer (Shimadzu, Kyoto, Japan) and agarose gel electrophoresis. RNA was reverse-transcribed using a MMLV kit (Evrogen, Moscow, Russia) with the recommended conditions.

Real-time PCRs were performed in triplicate using a SYBR Green I RT-PCR kit (Syntol, Moscow, Russia) and a CFX96 Real-Time PCR System (Bio-Rad, Hercules, CA, USA) with thermal cycling parameters: 95 °C for 3 min (one cycle) and then 95 °C for 15 s, 63 °C for 45 s, and 72 °C for 10 s (40 cycles). Real-time PCR primers for *Piwi* were designed using Primer Premier 5 software (Premier Biosoft International, Palo Alto, CA, USA). The primers were synthesized by Evrogen (Moscow, Russia). The *elongation factor 1α* (*Ef1α*) was used as a reference gene. The primers for *Ef1α* were as follows: Ef1α_F1 5′-AACACCGAGCCACCCTACAGC-3′ and Ef1α_R1 5′-CCGTCCCTCTTCCATCCCTT-3′. The data were processed using the software packages Bio-Rad CFX Manager 2.1 (version 1022.0523) and Microsoft Excel 2010 (version 14.0.7162.5000) and analyzed using the 2-ΔΔCt-method. Confidence intervals were calculated based on the standard deviation.

### 2.3. RNA Probe Synthesis

All cDNA samples used for qPCR were mixed and then used to amplify the cDNA fragment of 6 genes (*Piwi*, *MMP16*, *72 kDa type IV collagenase*, *tensilin3*, *Sox17*, and *Sox9/10*). The gene-specific primers for it were designed with Primer Premier 5 (Premier Biosoft International, Palo Alto, CA, USA) (Supplementary Table S1). DNA fragments were purified from the reaction by the Cleanup Mini Kit (Evrogen, Moscow, Russia). The resulting amplicons ranged in length from 600 to 800 b.p. and were sequenced with ABI Prism 3130xL (Applied Biosystems, Thermo Fisher Scientific, Waltham, MA, USA) to confirm the specificity of the PCR. Then, the amplicons were ligated into the pTZ19R vector (Thermo Scientific^TM^) and transformed into XL1-Blue *E. coli* competent cells (Evrogen) using heat shock. The transformed cells were cultured overnight on Luria–Bertani (LB) agar plates containing 100 μg·mL^−1^ ampicillin, 50 μM IPTG, and 40 μg·mL^−1^ x-Gal. White clones were selected and amplified with primers M13 F (5′–GTTGTAAAACGACGGCCAGT–3′) and M13 R (5′-CACAGGAAACAGCTATGACC–3′) to confirm the insertion. The selected white colonies grew in 3 mL LB medium containing 100 μg·mL^−1^ ampicillin for 16 h. The resulting cell cultures were used for the extraction of recombinant plasmids with a GeneJet Plasmid Miniprep kit (Thermo Scientific^TM^).

PCR with the resulting plasmids and one gene-specific and one M13 primer was carried out to obtain an insert with an RNA polymerase-binding site. RNA probe transcription was done by using a DIG-RNA-labeling mix (Roche), RNase inhibitor, 0.1-M DTT, T7, and Sp6 RNA polymerase. The reaction was incubated at 37 °C overnight. For each gene, there was synthesized antisense and a sense probe.

### 2.4. Whole Mount In Situ Hybridization

Regenerating anlagen of the AB and digestive system on 5–7 and 10 dpe were prefixed by the injection of 4% PFA into the body cavity. Next, the anlagen were cut out and postfixed in 4% PFA for 2 h at room temperature. After fixation, the anlagen were kept in methanol at −20 °C until use. The specimens were washed in PTW solution (0.1% Tween 20 in PBS) 3 × 5 min. Rinsed specimens were treated with 1% Triton X-100 for 10 min, then washed 3 × 5 min in PTW and processed with proteinase K (Evrogen) (100 μg·mL^−1^) for 8 min. The proteinase K activity was blocked by incubation in a glycine solution (2 mg·mL^−1^) 2 × 5 min. After the proteinase K treatment, the specimens were fixed in 4% PFA on PTW for 20 min. Prehybridization was done in a HYB solution (4× SSC, 50% formamide, Torula RNA (5 mg·mL^−1^) and heparin (0.15 mg·mL^−1^)) for 1 h at 65 °C. The RNA probes were diluted in HYB solution at a concentration 10 ng·mL^−1^ and denaturated for 10 min at 80 °C. Hybridization was done overnight at 65 °C. After hybridization, the specimens were washed 2 × 40 min in 50% formamide in 2× SSC, 2 × 20 min in 2× SSC, and 2 × 40 min in 0.2× SSC at 65 °C. To prevent nonspecific binding of the antibodies, the specimens were blocked in 5% normal goat serum in PTW for 2 h. Alkaline phosphatase-conjugated anti-DIG antibodies (Roche) were diluted 1:2000 in 2.5% normal goat serum in PTW. The specimens were incubated in anti-DIG antibodies overnight at 4°C. Next, the specimens were rinsed in PTW 3 × 5 min, 3 × 10 min, 3 × 15 min, and 2 × 30 min in a solution containing 0.1 M TRIS-HCl (pH 9.5), 0.1 M NaCl, and 0.1% Tween 20 and stained with BCIP/NBT solution (BCIP/NBT tablets; Roche).

After BCIP/NBT staining, the specimens were photographed under a Jenamed 2 (Carl Zeiss Jena, Oberkochen, Germany) light microscope equipped with a Nikon D1x digital camera (Nikon, Minato, Tokyo, Japan). Next, the specimens were incubated in 30% sucrose solution at 4 °C overnight and embedded in Neg-50 (Thermo Scientific Richard-Allan Scientific, Waltham, MA, USA) mounting medium for 30 min at −40 °C. The cryosections 50 μm thick were cut with a Thermo Scientific HM 560 CryoStar cryomicrotome (Thermo Fisher Scientific, Waltham, MA, USA) and examined under a Zeiss Axio Imager Z.2 (Carl Zeiss Jena, Oberkochen, Germany) and Keyence Biorevo BZ9000 light microscopes (Keyence, Osaka, Japan).

### 2.5. Sequence Searching and Phylogenetic Analysis

The sequences of the *E. fraudatrix* orthologs were found using Ensembl v103 (https://doi.org/10.1093%2Fnar%2Fgkx1098, accessed on 15 March 2021), Flybase vFB2021_02 (https://doi.org/10.1093/nar/gkaa1026, accessed on 15 March 2021), and Echinobase (https://doi.org/10.1093%2Fdatabase%2Fbax074, accessed on 15 March 2021) databases. Holothurian TIMP and MMP genes were found using the NCBI NR protein database, Echinobase, target domain searching in all the coding sequences of the species, and manual verification of the sequences with BLAST alignment against the NR and UniProt databases. All coding sequences were translated and checked for the full (more 90% of the length) main protein domain (Piwi, HMG_box, Peptidase_M10, etc.) with the HMMER v3.3 (https://doi.org/10.1371/journal.pcbi.1002195, accessed on 15 March 2021) and Pfam v33 databases (https://doi.org/10.1093/nar/gkaa913, accessed on 15 March 2021). The alignment was created using COBALT with the standard settings (https://doi.org/10.1093/bioinformatics/btm076, accessed on 15 March 2021). Gblocks v0.91 with nearly default settings (−b1 = 50% + 1, −b2 = 80%, −b3 = 7.5, −b4 = 5, and −b5 = n) was used for removing bad blocks from the alignment (https://doi.org/10.1093/oxfordjournals.molbev.a026334, accessed on 15 March 2021). Then, all the amino acids of the sequences in the alignment were replaced by the corresponding triplets from the original nucleotide sequences. For choosing the optimal settings of tree computing, IQ-TREE v2.1.1 was used (https://doi.org/10.1093/molbev/msaa015, accessed on 15 March 2021). Tree computing was performed by means of the IQ-TREE v2.1.1 tool with the best-selected model, 50,000 replicates of bootstrap, and NNI optimization (-bnni key). The sequences of the *E. fraudatrix* orthologs were found using the basic local alignment search tool (BLAST) program at the National Center for Biotechnology Information (http://www.ncbi.nlm.nih.gov/blast, accessed on 15 March 2021). The protein domains were identified by the Pfam (http://pfam.xfam.org, accessed on 15 March 2021) database search. The phylogenetic trees were inferred using the TVMe+G4 (*Piwi*), TIM2e+I+G4 (*tensillin*), and TIM2+F+I+G4 (*Sox*) models.

### 2.6. Immunocytochemistry

Rabbit polyclonal anti-PIWI antibodies were prepared by immunization with a peptide that included a highly conserved region located in the MID domain (VATKVAMQLNCKLGG). The peptide sequence was obtained based on the data from a *E. fraudatix* transcriptome analysis previously performed (contig number GHCL01004202.1) [31]. Peptide synthesis and primary polyclonal rabbit antibodies production were performed by the ALMABION Company (Russia).

Holothurians weighing 4.5–5 g were used in the experiment, 3 animals were used for each time point: intact animals (control); 1, 4, and 24 h post-evisceration (hpe); and 7 and 10 dpe. The detection of PIWI-positive cells was performed on smears of coelomic fluid cells prepared as previously described [11]. In addition, frozen tissue sections were used. Samples of the body wall and digestive tube were fixed in 4% paraformaldehyde in phosphate-buffered saline (PBS; pH 7.4, AMRESCO, Radnor, PA, USA) for 4 h and washed in three portions of PBS during the day. Pieces of the tissue were kept in a 15% sucrose solution in PBS (12 h) and embedded in the NEG 50TM medium (Thermo Scientific Richard-Allan Scientific, Waltham, MA, USA). Sections of the frozen material were made on a cryomicrotome HM 560 CryoStar (Thermo Fisher Scientific, Waltham, MA, USA).

Staining of the smears of coelomocytes and sections of frozen materials was performed using a similar technique. To prevent nonspecific binding of the primary antibodies, the samples were treated with a blocking buffer for 2 h (3% teleostean fish gelatin, SIGMA, 0.3 M glycine, and 0.5% Triton X-100 in PBS) and incubated with primary immune anti-PIWI antibodies diluted with 1% BSA in PBS at a ratio of 1:300 during 24 h at 4 °C. The smears were then washed with 1% BSA in PBS containing 0.02% TWEEN 20 (3 times for 10 min), incubated for 1 h at room temperature with Alexa 546-labeled secondary anti-rabbit antibodies (Molecular Probes), and diluted with 1% BSA in PBS at a ratio of 1:750. After washing three times in PBS, the preparations were embedded in a special DAPI-containing medium for staining nuclear DNA (Vectashield, Vector Laboratories). The material was analyzed using an LSM 780 laser confocal scanning microscope (Carl Zeiss, Germany).

The material was processed and analyzed at the Far Eastern Center of Electron Microscopy and the CKP “Primorsky Aquarium” (National Scientific Center of Marine Biology, Far Eastern Branch, Russian Academy of Sciences, Vladivostok, Russia).

## 3. Results

### 3.1. Identification and Characterization of DEGs of Piwi, MMP, TIMP, and Sox Orthologs

**Figure 1:**
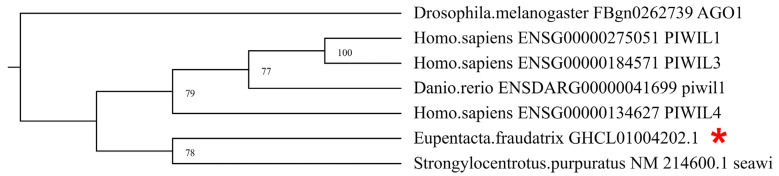
Phylogenetic tree showing the relationships of the Piwi sequence of *E. fraudatrix* (marked with the asterisk) with homolog proteins of other animals.

In the transcriptome of *E. fraudatrix*, a transcript was found that had a high degree of identity with the *Piwi* mRNA of *A. japonicus*. On the phylogenetic tree, it forms a single group with the Piwi of sea urchin (Figure 1). Based on the putative amino acid sequence of Piwi of *E. fraudatrix*, it was shown that its protein molecule contains three conserved domains: PAZ, MID, and PIWI. According to the transcriptome analysis, the *Piwi* expression during regeneration was not significantly different from the control values.

An analysis of the transcriptome of *E. fraudatrix* revealed a number of orthologs of DEGs of the MMP, TIMP, and Sox families. Among them, genes were selected, the expression of which is enhanced at the stages of transdifferentiation and formation of the gut luminal epithelium (5–7 and 10 dpe, respectively). Among the numerous *MMPs*, two genes were selected. The classification of the MMPs in echinoderms has not been developed [50], and it is impossible to determine the orthologs of these genes. According to the blast analysis, the first gene most likely encodes a proteinase close to 72-kDa type IV collagenase of holothurian *A. japonicus*. For this reason, it was defined as *Ef-72 kDa type IV collagenase* (GHCL01013204.1). The Ef-72 kDa type IV collagenase contains a signal peptide, a pro-peptide domain terminated by a cysteine switch, a catalytic domain, and three hemopexin-like repeats. The furin-activated motif is missing.

The second gene has a sequence close to *MMP16* of *A. japonicus*. In this connection, it was defined as *Ef-MMP16* (GHCL01010993.1). The Ef-MMP16 contains a signal peptide at the N-terminus, a pro-peptide domain with a cysteine switch, a furin-activated motif, a catalytic domain, and four hemopexin-like repeats.

**Figure 2:**
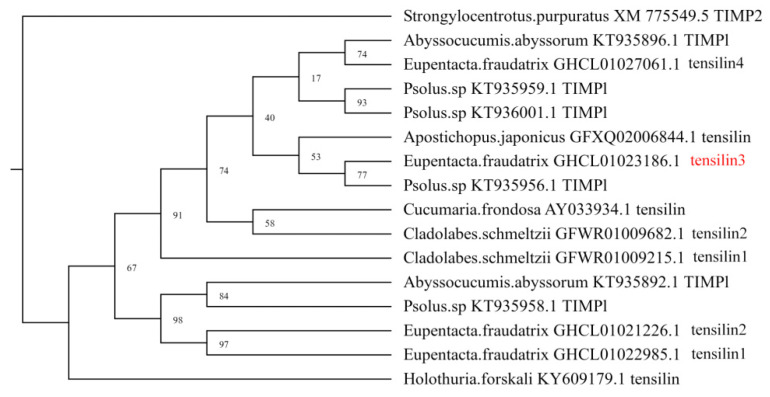
Phylogenetic tree showing the relationships of the tensilins of *E. fraudatrix* with homolog proteins of the other holothurians. The tensilin used in the study is marked with red.

The transcriptome of *E. fraudatrix* contains transcripts of at least seven *TIMP* genes. Of these, four genes encode specific proteins, tensilins. *Tensilins* represent a separate group of genes that have formed within the class Holothuroidea and are absent in other echinoderms [38,50]. According to the data of the transcriptome analysis, the *Ef-tensilin3* gene (GHCL01023186.1) exhibited the highest level of expression during gut regeneration. The maximum number of its transcripts was observed during the period of transdifferentiation (5–7 dpe). The phylogenetic analysis showed that all tensilins of the *E. fraudatrix* clustered together with the tensilins and TIMPs of the other holothurians (Figure 2). *Ef-tensilin3* encoded a protein with a structure typical of TIMPs. A NTR domain and 11 conservatively arranged cysteine residues were identified in the putative amino acid sequence of *Ef-tensilin3.*

**Figure 3:**
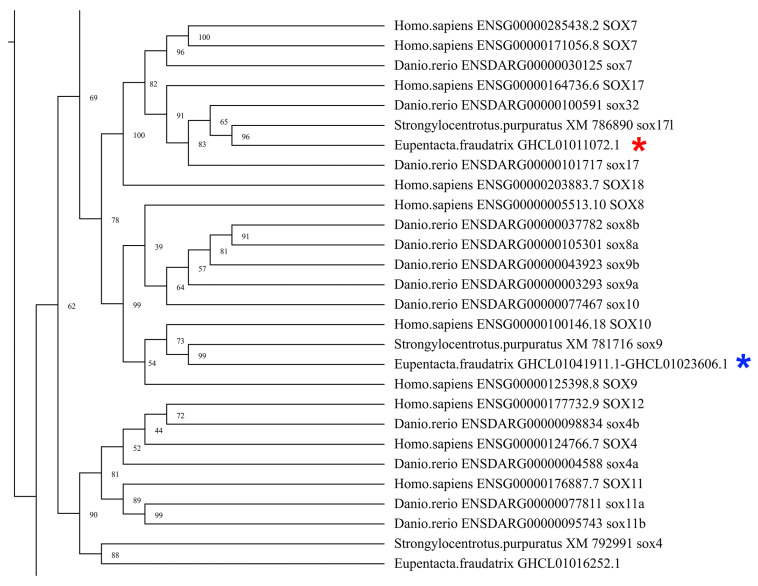
Part of the phylogenetic tree showing the relationships of *Sox* of the *E. fraudatrix* with homolog proteins of other animals. *Ef-Sox9/10* is marked with a blue star, and *Ef-Sox17* is marked with a red star.

An analysis of the *E. fraudatrix* transcriptome revealed transcripts of several genes of the Sox family with blast, such as *SoxB1*, *SoxD1*, *Sox4*, *Sox9*, *Sox17*, and *Sox21*. The orthologs of the *Sox9* and *Sox17* genes were the most active. The transcriptome contains two sequences (GHCL01041911.1 and GHCL01023606.1) coding parts of the same protein. This protein is clustered to the clade containing human *Sox9* and *Sox10* with strong bootstrap support (Figure 3 and Supplementary Figure S1). In this regard, these sequences of *E. fraudatrix* were designated as *Ef-Sox9/10*. The *Sox17* gene ortholog is located on the phylogenetic tree along with the *Sox17* genes of vertebrates (Figure 3 and Supplementary Figure S1). Thus, the corresponding gene of *E. fraudatrix* should be designated *Ef-Sox17.*

### 3.2. Spatial Distribution of Piwi-Positive Cells

**Figure 4:**
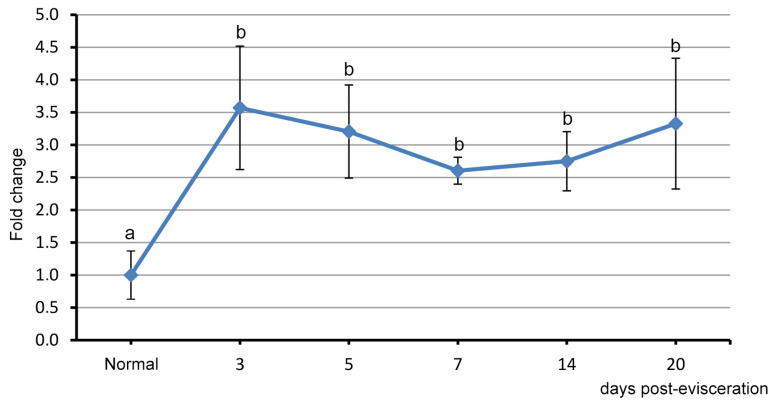
mRNA expression profile of *Piwi* during different days post-evisceration and in normal intestinal tissue in *E. fraudatrix*. Different lowercase letters indicate significant differences (*p* < 0.05). The data are reported as the means ± S.D. (*n* = 5).

Since the transcriptome analysis did not reveal differences in the number of *Piwi* transcripts between intact and regenerating animals, the expression of this gene in the gut anlage was further investigated using qPCR. It was shown that the number of *Piwi* transcripts changed in a wave-like manner during regeneration (Figure 4). In the early periods after evisceration, an increase in the *Piwi* gene expression occurs. After 3 dpe, the contents of its transcripts increased approximately 3.5 times compared to the control. Over the next few days, the expression level declined, reaching a minimum at 7 dpe. Then, a repeated increase in the expression was observed, which reached its maximum value after 20 dpe. Despite the period of decreased expression, its level throughout the observation period (20 dpe) remained significantly higher than the control values.

**Figure 5:**
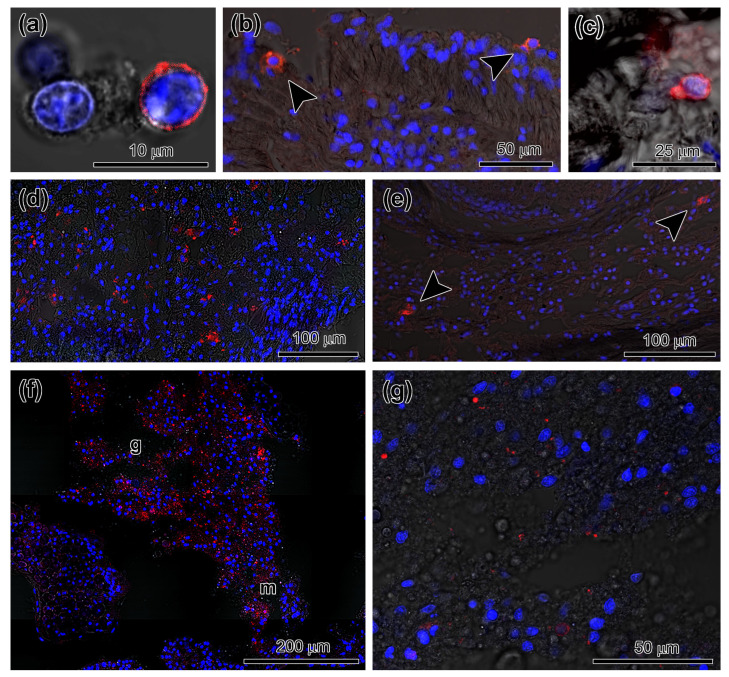
Localization of Piwi-positive juvenile cells in tissues of *E. fraudatrix*. (**a**) Labeled cell in the coelom at 4 hpe. (**b**) Labeled cells (arrowheads) in the coelomic epithelium at 4 hpe. (**c**) Labeled cell in the dermis of the body wall at 4 hpe. (**d**) Numerous labeled cells in the hypodermis at 4 hpe. (**e**) Rare-labeled cells (arrowheads) in the hypodermis at 24 hpe. (**f**) General view of the gut anlage at 7 dpe. (**g**) Labeled cells in the connective tissue of the gut anlage at 7 dpe. g, gut anlage; m, mesentery. Immunocytochemical staining with antibodies for the PIWI protein (red color) and DAPI-stained nuclear DNA (blue color).

In intact and regenerating holothurians, the Piwi protein was detected only in juvenile cells [11]. The Piwi-positive juvenile cells were found in various parts of the body of *E. fraudatrix*: coelomic fluid, mesothelium, connective tissue of the body wall, and internal organs (Figure 5a–c). After 4 hpe, many labeled cells were concentrated in the inner layer of the body wall, the hypodermis (Figure 5d). After 24 hpe, the contents of the labeled cells in both the coelomic fluid and in the tissues of the holothurian appeared to be significantly reduced [11]. Only single Piwi-positive cells were found in the connective tissue of the body wall (Figure 5e).

During the formation of the gut anlage, Piwi-positive juvenile cells were also detected in it. These cells were localized in the ECM and were absent in the developing luminal epithelium (Figure 5f,g). After 10 dpe, only single Piwi-positive juvenile cells were found in the intestine and in other organs.

### 3.3. Spatial Distribution of MMPs Transcripts

**Figure 6:**
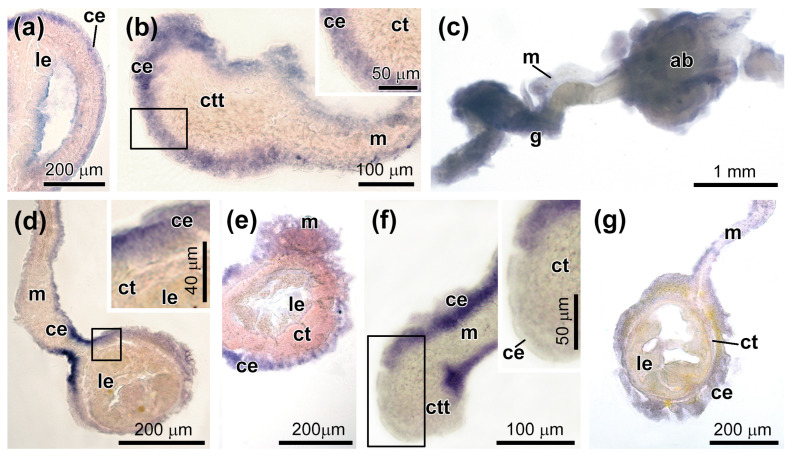
Expression of MMPs during gut regeneration. (**a**) *72 kDa type IV collagenase* expression in forming luminal epithelium of the gut on 5–7 dpe (histological section). (**b**) *72 kDa type IV collagenase* expression in the coelomic epithelium of the mesentery and connective tissue thickening of the gut anlage on 5–7 dpe (histological section). (**c**) *72 kDa type IV collagenase* expression in the posterior part of the gut on 10 dpe (whole mount). (**d**) *72 kDa type IV collagenase* expression in the coelomic epithelium of the mesentery and gut anlage on 10 dpe (histological section). (**e**) Expression of *MMP16* in the anterior part of gut anlage on 5–7 dpe (histological section). (**f**) Expression of *MMP16* in the coelomic epithelium of the posterior part of the mesentery and connective tissue thickening on 5–7 dpe (histological section). (**g**) Expression of *MMP16* in the coelomic epithelium of the mesentery and gut on 10 dpe (histological section). ab, aquapharyngeal bulb; ce, coelomic epithelium; ct, connective tissue; ctt, connective tissue thickening; g, gut; le, luminal epithelium; and m, mesentery; the insets in (**b**,**d**,**f**) show higher magnification views of the boxed areas.

**Figure 7:**
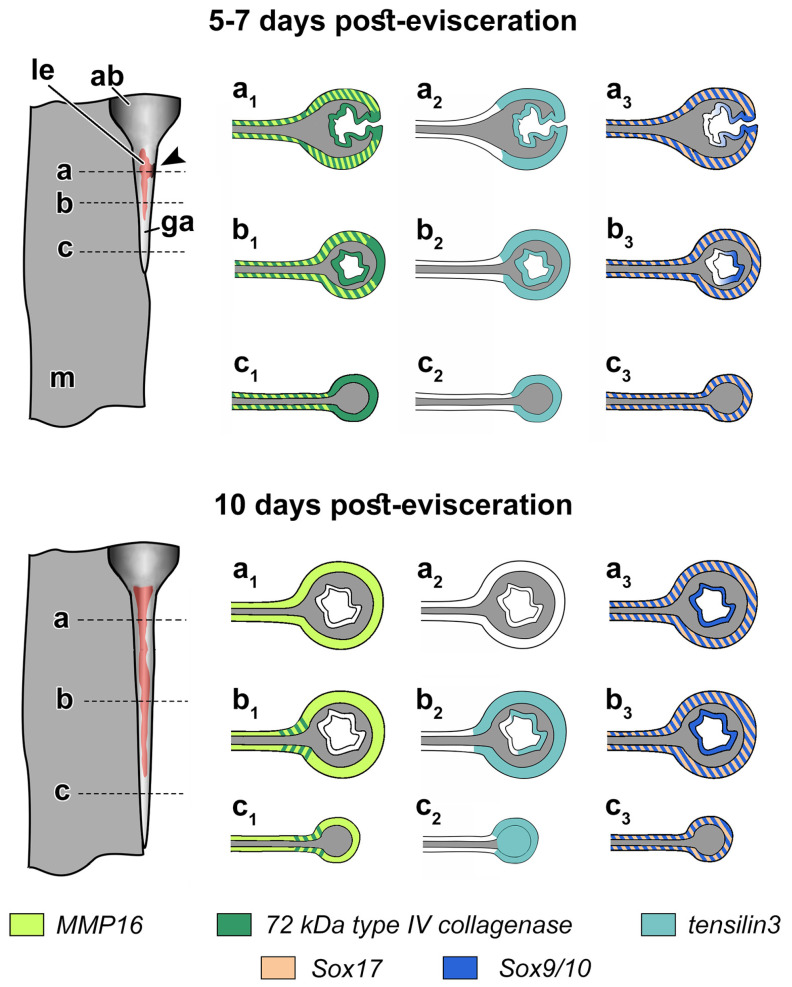
Scheme of spatial distribution of the *72 kDa type IV collagenase*, *MMP16*, *tensilin3*, *Sox9/10*, and *Sox17* transcripts on 5–7 and 10 dpe. (**a**–**c**): Dotted lines indicate the planes of the gut anlage cut and **a_1_**–**c_3_**: sections of the gut anlage on the corresponding planes; ab, aquapharyngeal bulb; ga, gut anlage; le, luminal epithelium of the gut; and m, mesentery; an arrowhead indicates a site of coelomic epithelium embedding.

After 5–7 dpe, the transcripts of *Ef-72 kDa type IV collagenase* were evenly distributed in the coelomic epithelium of the mesentery and gut anlage (Figure 6a,b and Figure 7). In addition, a small expression of this gene was found in the developing luminal epithelium of the gut (Figure 6a). At 10 dpe, *Ef-72 kDa type IV collagenase* transcripts were detected only in the posterior part of the growing digestive tube at a distance of 1 to 2 mm from AB (Figure 6c and Figure 7). In this case, the most intense expression was noted on the dorsal side of the gut, where it is attached to the mesentery (Figure 6d and Figure 7).

*Ef-MMP16* transcripts through 5–7 dpe were found in the coelomic epithelium of the mesentery and gut anlage, with the exception of their ventral part, where the expression of this gene was not detected (Figure 6e,f and Figure 7). Moreover, *Ef-MMP16* transcripts were absent in forming luminal epithelium (Figure 6e). After 10 dpe, the expression of *Ef-MMP16* decreased, and its transcripts were evenly distributed in the coelomic epithelium of the mesentery and gut (Figure 6g and Figure 7).

### 3.4. Spatial Distribution of Ef-Tensilin3 Transcripts

**Figure 8:**
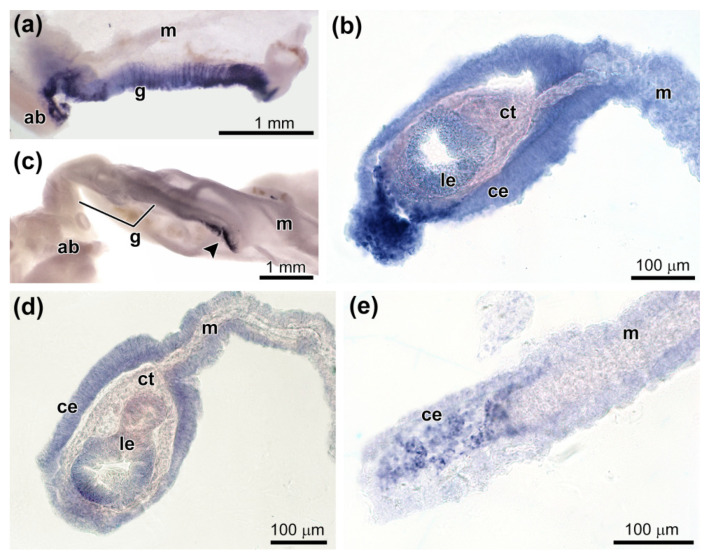
Expression of *tensilin-3* during gut regeneration. (**a**) *Tensilin-3* expression in the ventral part of the mesentery and gut anlage on 5–7 dpe (whole mount). (**b**) *Tensilin-3* expression in the ventral part of the gut anlage on 5–7 dpe (histological section). (**c**) *Tensilin-3* expression in the growing end of the gut on 10 dpe (whole mount). (**d**) *Tensilin-3* expression in the coelomic and luminal epithelia of the gut anlage on 10 dpe (histological section). (**e**) *Tensilin-3* expression in the ventral part of the growing end of the gut on 10 dpe (histological section). ab, aquapharyngeal bulb; ce, coelomic epithelium; ct, connective tissue; g, gut; le, luminal epithelium; and m, mesentery.

After 5–7 dpe, *Ef-tensilin3* transcripts were found in the coelomic epithelium of the mesentery and gut anlage (Figure 7 and Figure 8a,b). The most intense expression was located in the ventral part of the forming digestive tube. In the course of regeneration, the intensity of the expression decreased. In some individuals, during this period, the *Ef-tensilin3* transcripts formed only a narrow stripe, which expanded somewhat in the posterior part of the anlage. After 10 dpe, the highest expression of this gene occurred only in the growing tip of the gut (Figure 8c). Moreover, *Ef-tensilin3* mRNA was detected in the coelomic epithelium of the gut anlage and the ventral part of the mesentery (Figure 7 and Figure 8d,e). Furthermore, transcripts of the gene were found in the ventral part of the luminal epithelium (Figure 7 and Figure 8d).

### 3.5. Spatial Distribution of Ef-Sox9/10 and Ef-Sox17 Transcripts

**Figure 9:**
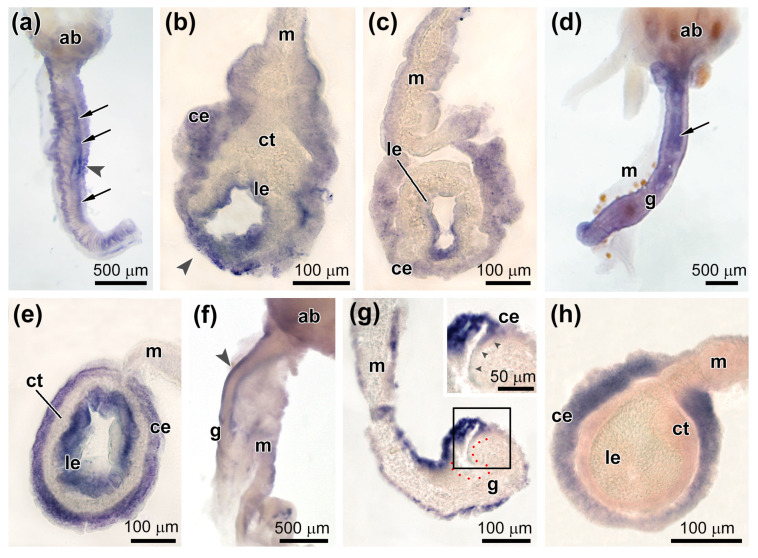
Expression of *Sox9/10* and *Sox17* during gut regeneration. (**a**) Expression of *Sox9/10* in the coelomic and luminal epithelia of the gut anlage on 5–7 dpe; an arrowhead indicates a site of coelomic epithelium embedding, and arrows show the luminal epithelium (whole mount). (**b**) Expression of *Sox9/10* in the coelomic and luminal epithelia of the gut anlage in the site of coelomic epithelium embedding (arrowhead) on 5–7 dpe (histological section). (**c**) Expression of *Sox9/10* in the luminal epithelium of the posterior part of the gut anlage on 5–7 dpe (histological section). (**d**) Expression of *Sox9/10* in the gut on 10 dpe (whole mount). (**e**) Expression of *Sox9/10* in the luminal epithelium in the middle part of the gut anlage on 10 dpe (histological section). (**f**) Expression of *Sox17* in the ventral part (arrowhead) of the gut anlage on 5–7 dpe (whole mount). (**g**) Expression of *Sox17* in the coelomic epithelium in the site of embedding on 5–7 dpe; red spots indicate the site of the epithelium embedding, and arrowheads in the insert show the embedding epithelium (histological section). (**h**) Expression of *Sox17* in the coelomic epithelium of the lateral and dorsal parts of the gut on 10 dpe (histological section). ab, aquapharyngeal bulb; ce, coelomic epithelium; ct, connective tissue; g, gut; le, luminal epithelium; and m, mesentery; the inset in (**g**) shows a higher magnification view of the boxed area.

After 5–7 dpe on the ventral side of the gut anlage, an area of intense expression of *Ef-Sox9/10* appeared (Figure 7 and Figure 9a). It was at a distance of about 500–600 μm from AB. This area corresponds to the area of immersion of the coelomic epithelium in the connective tissue of the gut anlage. The sections show that *Ef-Sox9/10* transcripts mark cells during immersion into connective tissue of the gut anlage (Figure 7 and Figure 9b). After immersion, the expression is retained in the cells. The ventral part of the luminal epithelium is labeled more intensively (Figure 9c). Small accumulations of *Sox9/10*-positive cells were also detected in the coelomic epithelium on the lateral sides of the gut anlage and in the mesentery (Figure 9b,c). In animals fixed at 10 dpe, the intensity of the *Ef-Sox9/10* expression increased. Its transcripts were localized both in the coelomic and in the luminal epithelium of the anterior gut anlage (Figure 7 and Figure 9d,e).

After 5–7 dpe, *Ef-Sox17* transcripts were detected in the coelomic epithelium of the gut anlage (Figure 7 and Figure 9f). At the same time, their greatest concentration was noted at the site of immersion only in surface cells (Figure 9g). With the development of the digestive system (10 dpe), the expression of *Ef-Sox17* was retained only in the coelomic epithelium; the products of this gene were absent in the luminal epithelium of the gut (Figure 7 and Figure 9h).

## 4. Discussion

One of the important aspects of the study of regeneration is the question of the origin of the cells from which the lost organs are formed. In many cases, different types of stem/progenitor cells participate in regeneration [51]. The presence of stem cells, with the exception of progenitor cells of coelomocytes (juvenile cells) [10,11] and primordial germ cells, has not been established for echinoderms [12,13]. Nevertheless, there are some papers describing the participation of stem cells in regeneration in these animals [24,25,26]. In this regard, we tried to identify the role of Piwi-positive cells in the formation of the digestive system in *E. fraudatrix*.

During evisceration, holothurians lose a significant volume of coelomic fluid together with the cells it contains. The restoration of the cellular composition of the coelomic fluid occurs in the absence of proliferation of the rest of the coelomocytes, which suggests that *E. fraudatrix* has an external reserve subpopulation of progenitor cells located in certain tissues and organs of the animal [10,11]. It is assumed that the niches of stem cells of coelomocytes in echinoderms can be various epithelia, connective tissue, and the nervous system [52,53]. In this regard, our data on the localization and dynamics of Piwi-positive juvenile cells in the tissues of regenerating holothurians are of particular interest. An increase in the number of the cells in the connective tissue of the body wall after 4 hpe may indicate that *E. fraudatrix* has a pool of progenitor cells in this place.

Our research has shown that there are Piwi-positive juvenile cells in the gut anlage. However, they are located only in the ECM and, obviously, do not participate in the formation of the luminal epithelium of the digestive tube. Apparently, these labeled cells are descendants of juvenile cells that migrated from the body wall and are at the initial stages of differentiation. The increase in *Piwi* expression during the gut regeneration, according to qPCR data, probably reflects the same process. It is known that, after rising in the first hours after evisceration, the number of juvenile cells decreases but remains at a high level for a long time [11,12]. Their increased content in the coelom and tissues of holothurians during the restoration of the cellular composition of coelomic fluid probably also affects the number of *Piwi* transcripts, which are detected in the gut using qPCR.

A feature of the regeneration of the digestive system in *E. fraudatrix* is the presence of transdifferentiation during the formation of the luminal epithelium of the anterior part of the gut [47]. The transformation of one cell type into another is a complex process involving a large number of different genes [28,31]. However, the mechanism of gut regeneration includes not only transdifferentiation but also the formation of the base of the organ (connective tissue thickening), ECM remodeling, dedifferentiation, proliferation, and migration of many types of cells [48,49,54]. In all these processes, various proteinases play an important role, including MMPs [50,55,56]. It was previously shown that the inhibition of MMP activity led to a slowdown or even complete cessation of regeneration in holothurians [34,35,57,58]. This study has confirmed the important role of MMPs in the formation of the gut.

The studied proteinases by their domain structure can be attributed to different groups of MMPs [50,59]. Ef-72 kDa type IV collagenase is an archetypal MMP, because it does not contain a furin-activated motif, and Ef-MMP16 is a furin-activatable MMP. Since echinoderm MMPs actively diverged and duplicated [60], it is impossible to identify the orthologs of these genes in mammals, and, therefore, no analogies can be drawn between their properties.

An analysis of the spatial distribution of the transcripts showed that these proteinases appear to have different functions. During the period of transdifferentiation (5–7 dpe), the mRNA of *Ef-72 kDa type IV collagenase* is found in the gut anlage in cells of both the coelomic and luminal epithelia. Then, after the formation of the digestive epithelium (10 dpe), the expression of this gene is shifted to the dorsal side of the gut to the region of its junction with the mesentery. This distribution may indicate that Ef-72 kDa type IV collagenase is involved in the ECM remodeling and in cell migration, including their immersion in the connective tissue of the anlage and movement there. In the early stages of gut regeneration in holothurians, there is an active migration of cells along the mesentery and their immersion into the connective tissue [47]. Ef-72 kDa type IV collagenase is probably required for the degradation of ECM proteins and facilitating cell movement. In the process of the formation of the digestive system in holothurians, the gut mesentery lengthens [60]. The expression of this gene at the site of attachment of the digestive tube to the mesentery after 10 dpe seems to indicate that growth occurs precisely in this place.

The *Ef-MMP16* transcripts at both of the observed stages of regeneration are localized exclusively in the coelomic epithelium. Moreover, they are absent from the ventral side of the anlage, where the immersion and transdifferentiation of cells occurs. It is possible that this proteinase is involved in the regulation of migration and/or proliferation of coelomic epithelial cells. Similar data were obtained on the gut regeneration in *A. japonicus*. This holothurian activates two MMPs with different functions [36]. These proteinases are expressed only in the coelomic and luminal epithelia of the gut anlage. These results coincide with our data. The similarity in the distribution of *MMPs* transcripts in holothurians confirms the previously made conclusion that the main mechanism of regeneration of the digestive system in the animals is epithelial morphogenesis [27,28].

TIMPs are natural inhibitors of MMPs [61]. During regeneration, TIMPs can act as antagonists of proteinases for the more precise regulation of morphogenesis. In *E. fraudatrix*, of all the TIMP genes, *Ef-tensilin3* was the most active. Tensilins are a separate group of TIMP-like proteins of holothurians [38,50]. Previously, it was assumed that tensilins were involved in the mechanisms of regulation of the mechanical properties of the connective tissue in echinoderms [37,39]. The expression of *Ef-tensilin3* in the gut anlage indicates that these proteins may have other functions as well. Since tensilins are TIMP-like proteins [38], Ef-tensilin3 may be involved in inhibiting the activity of MMPs. It is well-known that any morphogenesis, including regeneration, necessarily requires a complex interplay and equilibrium between connective tissue degradation and maintenance [62]. The simultaneous expression of the MMP and TIMP genes is likely to reflect the fine regulation of the ECM remodeling process during gut regeneration. In holothurians, during the formation of the gut anlage, collagen is synthesized and accumulated in the ventral edge of the mesentery [35,63]. Probably, the blocking of proteinase activity is necessary for the stabilization of the ECM and the development of the connective tissue base of the digestive tube. Interestingly, the sites of expression of the *Ef-MMP16* and *Ef-tensilin3* genes after 5–7 dpe are somewhat opposite to each other. During this period, *Ef-MMP16* transcripts are absent in the ventral part of the gut anlage—that is, exactly where the highest *Ef-tensilin3* expression is observed. These data suggest that Ef-tensilin3 may be an inhibitor of the Ef-MMP16 proteinase.

It is known that TIMPs can perform functions other than inhibiting proteinases. For example, in mammal TIMP-1, interacting with MT1-MMP helps to activate pro-MMP2 [64]. This mechanism stimulates cell migration during tumor metastasis and invasion. In addition, TIMPs show cell growth promoting activity and can modulate cell apoptosis [65,66,67,68]. Moreover, TIMP-1 is able to bind to CD63 and integrins and regulate cell survival and polarization [69,70]. In this regard, tensilins and other TIMPs may be involved in similar processes in holothurians. Apoptosis is observed during the digestive system formation in these animals [71]. Furthermore, a transcriptome analysis showed that gut regeneration in holothurians is accompanied by a change in the expression level of *integrins* [72].

Our research has shown that the genes *Ef-Sox9/10* and *Ef-Sox17* may participate in regulation of the digestive system regeneration in holothurians. The expression of both genes through 5–7 dpe is found at the site of immersion of coelomic epithelium cells and the formation of gut luminal epithelium. This could mean that Ef-Sox9/10 and Ef-Sox17 can play a role in transdifferentiation. As in the case of MMPs, these genes may differ in their functions. Since *Ef-Sox17* mRNA is detected at the site of immersion only in surface cells, this gene is probably involved in the regulation of the initial stages of transdifferentiation. *Ef-Sox9/10* transcripts are found not only in the coelomic epithelium but also in submerged cells and the developing luminal epithelium. This may mean that *Ef-Sox9/10* controls the process of switching mesodermal cells to the enterocyte phenotype. Our results agree with data on other animals in which orthologs of these genes are involved in endoderm specification and digestive system regeneration [41,42,73]. In particular, in the early development of mammals, the progenitor cells of the intestine express *Sox9* [74]. Further, SOX9 specifies the cell fate and differentiation in many cell lineages, including gut cells [75].

Simultaneously with their participation in transdifferentiation, both of these genes, possibly, perform other functions. Throughout the period under consideration, they were expressed in the coelomic epithelium of the digestive tube and mesentery. Possibly, *Ef-Sox9/10* and *Ef-Sox17* are also involved in the redifferentiation of myoepithelial cells and the formation of gut musculature. In mammals, orthologs of these genes are involved in the specification of mesodermal cells [76,77].

## 5. Conclusions

Our study revealed the presumptive location of juvenile cells, which are progenitor cells of coelomocytes in holothurians. Juvenile cells are localized in the loose connective tissue layer of the body wall (hypodermis). In the first hours after the loss of coelomocytes and coelomic fluid, they are activated, which is shown in the expression of the *Piwi* gene and proliferation. Like any echinoderm coelomocytes, which are called “wandering cells”, they are able to move in the ECM and epithelia of various body tissues. This probably explains the presence of Piwi-positive juvenile cells in the organs of holothurians, including the gut anlage. However, these cells are not involved in the formation of the luminal epithelium of the digestive tube.

Our data support the important role of ECM remodeling in regeneration in the echinoderms. After evisceration, *E. fraudatrix* expresses a large number of *MMPs* and *TIMPs*. Each of these genes has an individual expression profile and, accordingly, functions. Interestingly, among the *TIMPs*, the *Ef-tensilin3* gene exhibited the highest expression. This indicates that the functions of tensilins in holothurians may not be limited to the control of the mechanical properties of connective tissue. These TIMP-like proteins can be involved in the regulation of morphogenesis.

The genes of the Sox family, *Ef-Sox9/10* and *Ef-Sox17*, in *E. fraudatrix* are possibly involved in the transdifferentiation of coelomic epithelial cells. Their transcripts mark the cells that plunge into the connective tissue of the gut anlage and give rise to enterocytes. At the same time, the functions of *Ef-Sox9/10* and *Ef-Sox17* may differ in transdifferentiation. *Ef-Sox9/10* probably controls the switching of mesodermal cells to the enterocyte phenotype, while *Ef-Sox17* is involved in the regulation of the initial stages of transdifferentiation.

## Supplementary Materials

The following are available online at https://www.mdpi.com/article/10.3390/genes12081292/s1: Figure S1: Phylogenetic tree showing the relationships of *Sox* of the *E. fraudatrix* with the homologous proteins of other animals. Table S1. Sequences of gene-specific PCR primers.
