# Supplementary material for: Expression of Piwi, MMP, TIMP, and Sox during Gut Regeneration in Holothurian Eupentacta fraudatrix (Holothuroidea, Dendrochirotida)
Source: Genes (Basel). 2021 Aug 23;12(8):1292. doi: 10.3390/genes12081292 (PMC8391186; doi:10.3390/genes12081292)
Supplement: Supplementary file 1 [file genes-12-01292-s001.zip › Table S1. Sequences of gene-specific PCR primers.pdf]

Table S1. Sequences of gene-specific PCR primers

Primer	Sequence
Piwi F	5′ TAGAATACACCCAGAAGTGGCAGG 3′
Piwi R	5′ CTTCAGTGGCACAGTGGAGACAA 3′
MMP16 F	5′ GCCTTGGCTTTCATCTGCG 3′
MMP16 R	5′ ACCCCCTGAGACTTACCTCT 3′
72 kDa type IV collagenase F	5′ CGAATGATGATGTCCGAAAT 3′
72 kDa type IV collagenase R	5′ ACTGACAGTAGCGCGTGC 3′
tensilin3 F	5′ GTAGCCATTGCTTTACGACGC 3′
tensilin3 R	5′ CTGTATCTTCTGACGCTCCTGC 3′
Sox 17 F	5′ GGCATCCAAATCAACTCATCCA 3′
Sox 17 R	5′ CCTATGGTCCGCTATCTAAGTC 3′
Sox 9/10 F	5′ ACGGATACGACTGGTCAACGA 3′
Sox 9/10 R	5′ TTACCTGTCGCCTTCCGCTA 3′
