# Supplementary figures and images for: Expression of Piwi, MMP, TIMP, and Sox during Gut Regeneration in Holothurian Eupentacta fraudatrix (Holothuroidea, Dendrochirotida)
Source: Genes (Basel). 2021 Aug 23;12(8):1292. doi: 10.3390/genes12081292 (PMC8391186; doi:10.3390/genes12081292)

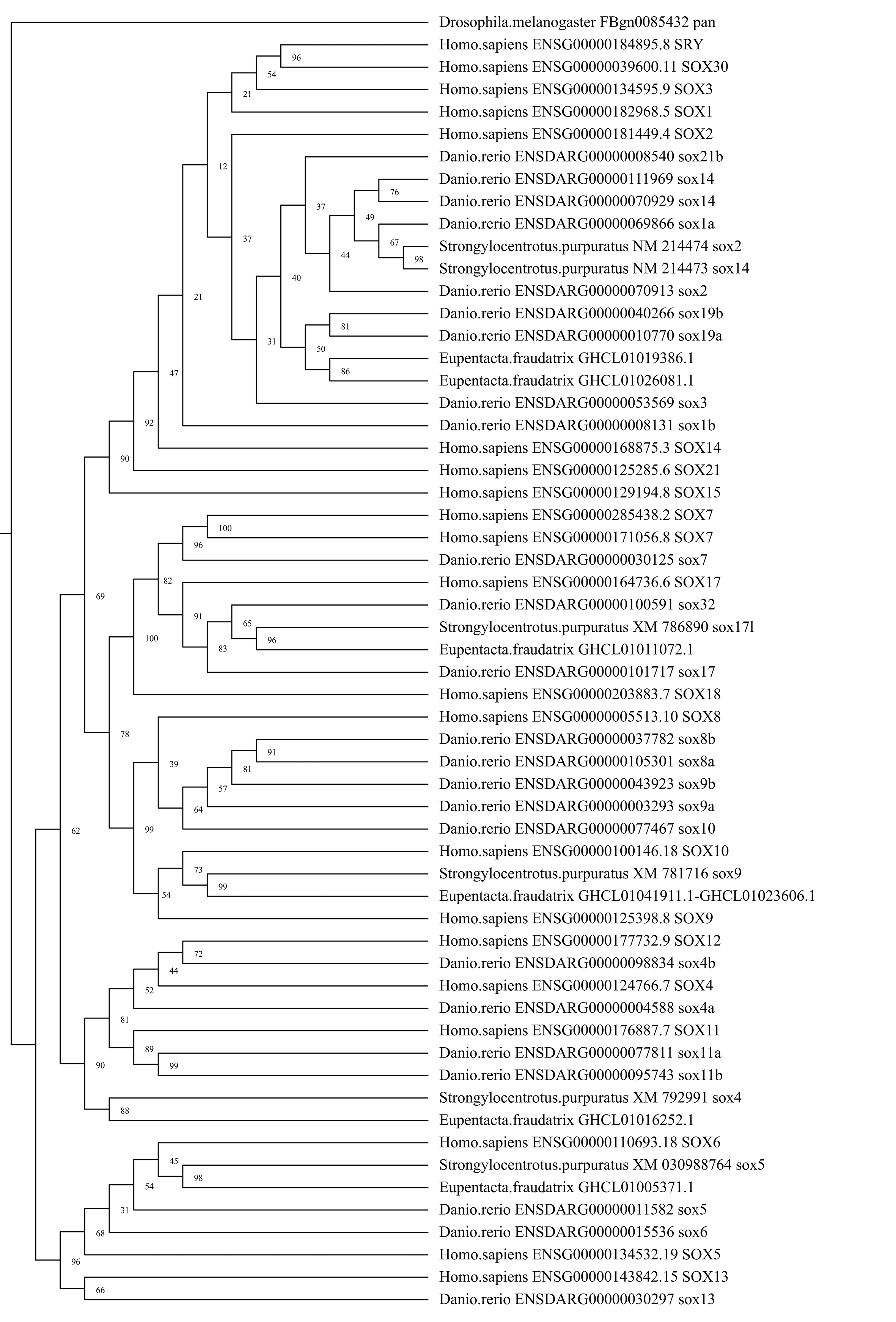

Supplement: Supplementary file 1 [file genes-12-01292-s001.zip › Figure S1. Phylogenetic tree.tif]
